# The Correlation between Microalbuminuria and Thyroid Nodules in Type 2 Diabetic Mellitus

**DOI:** 10.1155/2022/2789279

**Published:** 2022-03-07

**Authors:** Can Cao, Zhao-Li Cui, Juan Miao, Jing-Xin Zhou, Xiao-Nan Wang, Jian Jin

**Affiliations:** Department of Nephrology and Endocrinology, Dongzhimen Hospital, Beijing University of Chinese Medicine, Beijing, China

## Abstract

**Objective:**

To investigate the correlation between thyroid nodules and microalbuminuria in type 2 diabetic mellitus.

**Methods:**

A total of 270 patients with type 2 diabetes at Tongzhou Branch of Dongzhimen Hospital were enrolled in this retrospective study. Data were collected from the inpatient electronic files between January 2018 and January 2020. The laboratory indexes of the two groups (thyroid nodule group with 172 cases and control group including 98 cases without thyroid nodules) were statistically analyzed by binomial logistic regression analysis and Spearman correlation analysis.

**Results:**

The proportion of microalbuminuria (MAU) in the thyroid nodule group was larger than that in the control group. Age, serum TT4, and FT4 in the thyroid nodule group were significantly higher compared with the control group. The binary logistic regression analysis indicated that age, sex, FT4, and MAU were the risk factors for thyroid nodule in T2DM patients. Spearman correlation analysis showed that the thyroid nodule was significantly positively correlated with MAU, age, FT4, and TT4.

**Conclusions:**

MAU might be an independent risk factor for thyroid nodule in type 2 diabetic mellitus.

## 1. Introduction

Thyroid nodules (TNs) are a common clinical problem encountered in an endocrine practice, with an estimated prevalence on the basis of ultrasound that ranges from 20% to 76% in the general population [1]. In addition, type 2 diabetes mellitus (T2DM) is also a common disease in endocrinology, which is frequently coexisting with thyroid nodules. In recent years, the relationship between T2DM and TNs has been investigated quite intensively. Some evidence indicates that the incidence of thyroid nodules is significantly increased in T2DM patients in China [2]. Insulin resistance (IR), one of the characteristics of T2DM, has been demonstrated to be a risk factor for TNs in patients with T2DM [3]. Thyroid function in T2DM patients may be also closely associated with microalbuminuria (MAU), which is an early manifestation and one of the important prediction and prognosis of diabetes nephropathy [4]. FT3 is reported to negatively correlate with microalbuminuria in T2DM patients independent of other risk factors [5]. Meanwhile, other study has shown that thyroid stimulating hormone (TSH) levels are negatively associated with TN formation [6]. However, the association between microalbuminuria and thyroid nodules in T2DM has not been clarified. In the present study, we aimed to investigate the correlation between TN and MAU in patients with T2DM.

## 2. Materials and Methods

### 2.1. Subjects

The study was approved by the Medical Ethics Committee of the Dongzhimen Hospital, Beijing University of Chinese Medicine. Since data were collected from the inpatient electronic files in this retrospective study, no informed consent was signed. All participants aged 18 years or older who diagnosed with T2DM according to the guidelines for prevention and treatment of type 2 diabetes in China (2020) [7] from January 2018 to January 2020 at Tongzhou Branch of Dongzhimen Hospital were identified and included. According to the Chinese guidelines for diagnosis and treatment of diabetic kidney disease (DKD) [8], the diagnosis of MAU included patients with a definite diagnosis of DKD in the past or those who had over twice positive results in three times microalbuminuria tests within six months. The diagnostic criteria for TNs referred to the guidelines for the diagnosis and treatment of thyroid nodules and differentiated thyroid cancer [9]. Exclusion criteria included incomplete clinical data, the history of thyroid surgery, the history of levothyroxine tablets or other antithyroid drugs, TSH＜0.56 uIU/ml, severe liver disease and renal failure, infected, acute diabetic complications, hypoglycemia, and acid-base disturbance within 2 weeks.

### 2.2. Laboratory Assays and Ultrasound Measurements

Blood was drawn after an overnight fast at least 8 h. Glycated hemoglobin typeA1c (HbA1c, %), fasting plasma glucose (FPG, mmol/L), triglycerides (TG, mmol/L), total cholesterol (TC, mmol/L), low-density lipoprotein (LDL, mmol/L), high-density lipoprotein (HDL, mmol/L), serum creatinine (Scr, *µ*mol/L), blood urea nitrogen (BUN, mmol/L), and uric acid (UA, *µ*mol/L) were measured using the automatic biochemical analyzer (Beckman-DXC800, America). The thyroid function evaluation index including the free FT4 (reference range 5.44–11.85 ug/dL), free FT3 (reference range 0.66–1.61 ng/mL), serum thyroid stimulating hormone (TSH, reference range 0.56–5.91 uIU/mL), TT4 (reference range 5.44–11.85 ug/dL), TT3 (reference range 0.66–1.61 ng/mL), thyroid peroxidase antibody (TPO, reference range 0.0–9.0 IU/mL), and thyroglobulin antibody (TGAB, reference range 0.0–4.0 IU/mL) was determined by the automatic immune analyzer (Beckman-DXC800i, American).

The urine samples were collected and then used to detect urine microalbumin (UALB, mg/L). The collection of urine samples should meet the following requirements: naturally excrete and clean voided midstream urine, in which women should avoid collecting urine samples during menstruation and men should avoid the pollution of semen and prostate fluid, urine is collected in disposable clean and dry containers, the first morning urine of the day that were tested usually within 60 minutes.

The data of TNs were obtained from ultrasound reports. With reference to Mei-q Liu [10] and ACR TI-RADS classification [11], formulated the scoring rules of nodules: echogenicity, shape, margins, and echogenic foci. Each of the items was scored 2 points, respectively, and the total score ≥4 was defined as the ultrasonic high score. All ultrasound examinations were performed by radiologists blinded to the treatment and utilized a 6–15 MHz/50 mm linear probe (Logiq E-9, GE Medical Systems, WI, USA).

### 2.3. Statistical Analysis

Continuous variables in this study were skewed data (Kolmogorov–Smirnov test: *P* < 0.1 for each), and they would be expressed as median (interquartile range). Mann–Whitney *U*-tests were used to compare the differences in the clinical characteristics between the TN group and the control groups. The *χ*^2^ test was used for nonparametric variables. In the multivariate binary logistic regression analysis, the predictors of increased TNs were selected based on both clinical and statistical significance. Spearman correlation analysis was used to evaluate the correlation between TNs and clinical data. The predictors of TNs include age, gender, thyroid function evaluation index (FT3, TT4, and FT4), and MAU. For patients with a definite diagnosis of DKD, the results of the first time UALB after admission were used for analysis, and the average value of three times UALBs for others was analyzed. The data were analyzed by SPSS software (Statistical Package for the Social Sciences, version 25.0, Chicago). *P* < 0.05 is considered statistically significant.

## 3. Results

The clinical characteristics of the subjects are given in Table 1. The present study included a total of 270 T2DM cases (172 patients in the TN group and 98 patients in the control group). The two groups were well balanced at baseline regarding all the biochemical parameters including HbA1c, FPG, TG, TC, SCR, BUN, LDL, HDL, and UA. In our study, the incidence of TNs in males was lower than that in females (56.6% versus 68.9%, *P*=0.004). Age, serum TT4, FT4, and UALB were significantly higher in the TN group than in the control group (57 (50, 65) versus 50 (44, 60), *P* < 0.001; 8.34 (7.45, 9.31) versus 7.78 (7.03, 8.81), *P*=0.013; 0.89 (0.82, 0.98) versus 0.86 (0.77, 0.95), *P*=0.038; 22.00 (10.0, 67.55) versus 11.15 (10.0, 25.8), <0.001, respectively). The incidence of MAU in the TN group was significantly increased compared with the control group (44.8% versus 22.4%, *P* < 0.001). In all the enrolled patients with MAU, ratio for the use of renin-angiotensin system inhibitors (RASi) expressed no statistically significant difference between TN and non-TN patients (51.9% versus 31.8%, *P*=0.095). TSH, TT3, TGAB, and TPO levels had no significant difference between the two groups (Table 1).

The binary logistic regression analysis demonstrated that age, sex, FT4, and MAU remained as the risk factors for TN in T2DM patients (*β* = 0.033, *P*=0.005; *β* = −0.862, *P*=0.003; *β* = 2.462, *P*=0.014; *β* = 1.128, *P* < 0.001, respectively) (Table 2). Conversely, TT4 did not affect TN (Table 2).

Spearman correlation analysis showed that TNs were significantly positively correlated with MAU and UALB (rs value: 0.223, *P* < 0.001; rs value: 0.213, *P* < 0.001, respectively). Also, a positive correlation between TN and age, sex, FT4, and TT4 was observed (rs value: 0.229, *P* < 0.001; rs value: 0.177, *P*=−0.004; rs value: 0.127, *P*=0.038; rs value: 0.152, *P*=0.012). However, TN did not exhibit any correlation with other thyroid function evaluation indices (Table 3).

We further analyzed the correlation between ultrasound features and MAU in the TN group. In this study, the TN group encompass MAU 77 (44.8) and non-MAU 95 (55.2). The ultrasound features of thyroid nodules such as echogenicity, shape, margins, echogenic foci, and high scores of ultrasound were similar between the MAU groups and the non-MAU group (Table 4).

## 4. Discussion

In this retrospective study, we found that incidence of TN in patients with T2DM was statistically significantly increasing in females and older ages. The above results are consistent with many previous research conclusions [3]. In a previous study, it indicated that the thyroid size and volume of thyroid nodules significantly increased during pregnancy [12]. Another study also demonstrated that female as the risk factor for TN might be caused by the high demand of thyroxine and the periodic changes in the secretion of hormones during menstruation, pregnancy, and lactation [13]. The thyroid nodules have also been reported to increase with age, which is considered as a risk factor for thyroid cancer [14].

It has been shown that serum TSH played a major role in the formation of TN, while FT4 was not a risk factor for TN [15]. However, our finding displayed that serum FT4 and TT4 were significantly higher in the TN group than in the control group. Strong correlations between TN and lower serum FT3 and higher FT4 concentrations were shown in a consistently previous study [3]. Yalakanti [16] indicated that patients with T2DM might have mutations in the gene encoding type II 5′-deiodinase, which caused a decrease in enzyme activity, and lead to a significant reduction of FT3. The increase of FT4 supported the hypothesis that decrease of enzyme activity resulted in the reducing the conversion of T4 to T3. The logistic regression analysis revealed that FT4 was a risk factor for TN in T2DM patients.

Our results revealed that the abnormal rate or value of MAU in the TN group of T2DM patients was significantly higher than that in the control group (*P* < 0.05). In addition, multivariate binomial logistic regression analysis and Spearman correlation analysis exhibited that MAU was positively correlated with TN (*P* < 0.05), while the correlations between TN and FT4 and TT4 were also positive. Since more use of RASi in MAU patients, the ratio for the use of RASi was increased in the TN group compared with the control group. We further performed statistical analysis on all the enrolled patients with MAU, which showed that there was no statistically significant difference in the ratio for the use of RASi between thyroid nodule and nonthyroid nodule patients in this study. Therefore, the use of RASi had no statistical effect on the correlation between MAU and TN. In addition, red blood cells and white blood cells in the urine may affect the results of urine protein testing. We also conducted statistical analysis separately, regarding the difference between red blood cells and white blood cells in the two groups. The results showed that there was no statistically significant difference in the red blood cells and white blood cells in the urine between the two groups.

In addition, we observed a correlation between TI-RADS classification and MAU in thyroid nodules. For the two groups with TI-RADS classification scores >4 and ≤4, the incidence of MAU was similar. So, we identified that there was no significant difference of MAU between benign and malignant TNs.

Previous studies have shown that the occurrence of TNs was closely related to chronic inflammation attributed to the increase in proinflammatory factors [17]. Liu CL [18] found that the inflammatory indexes of patients with thyroid nodules were higher than the normal range. Inflammation was considered as a risk factor of TN due to its indirect effect [19]. Meanwhile, serum FT4 was demonstrated to significantly correlate with inflammatory marker TNF-*α* in patients with T2DM [20]. Our study pointed out that FT4 was as a risk factor for the thyroid nodule in T2DM patients. Therefore, there might be a close relationship between TNs and chronic inflammation. Additionally, type 2 diabetes is a considered to be chronic low-level inflammatory state. Systemic oxidative stress and local inflammatory damage not only promote the development of T2DM [21] but also play a key role in the pathogenesis of diabetic nephropathy [22]. Diabetic nephropathy is caused by multiple mechanisms, resulting in renal functional decline and structural damage. Among these pathogenic mechanisms, inflammation causes the accumulation of extracellular matrix of the glomerular mesangium, which leads to thickening of the basement membrane and continuous proliferation of vascular endothelial cells, which promote renal interstitial fibrosis and glomerular sclerosis [22]. Interleukin 1 (IL-1), IL-6, IL-18, and tumor necrosis factor (TNF) are considered the crucial inflammatory cytokines in diabetic nephropathy [23]. IL-6 and TNF-*α*, which are highly expressed in many inflammatory diseases, significantly increased in T2DM patients [24, 25]. Shikano [26] suggested that IL-6 promotes the growth of mesangial renal cells and seem to be a good indicator of diabetic nephropathy, especially the urinary levels. Also, TNF-*α* can directly lead to kidney injury, apoptosis, and necrosis, thereby producing cytotoxic effects on the kidney. TNF-*α* could change glomerular hemorheology and is associated with renal hypertrophy in early stage of diabetic nephropathy [23, 27]. In a word, proinflammatory factors have an impact on the occurrence and development of diabetic nephropathy and the formation of TNs. T2DM causes the aggregation and release of inflammatory factors that damage the kidneys and lead to TN formation. In this process, MAU is an early predictor of diabetic nephropathy, which reflects the balance between glomerular filtration and renal tubular reabsorption of albumin [28, 29]. Therefore, we infer that the underlying mechanism of positive correlation between MAU and TNs might be attributed to inflammation.

## 5. Conclusion

In summary, it is necessary to explore the risk factors and predictive factors of TNs because of the high incidence and relatively high risk of TNs in patients with T2DM. Abnormal urinary microalbumin in patients with T2DM may be an important indicator for screening thyroid nodules. In clinical work, we should pay attention to thyroid ultrasonography, when microalbumin is found in urine. Female, advanced age, and abnormal T4 values are also independent risk factors for TNs in patients with T2DM. Therefore, in clinical practice, health education and regular physical examinations should be focused on MAU, women, elderly, and people with thyroid dysfunction, among patients with T2DM. It is very important to focus on the screening of thyroid nodules, early detection and early intervention, and improve the quality of life of patients, especially in the population with abnormal thyroid function and microalbuminuria. In addition, there may still be some limitations in this study. First, serum samples were not retested, which may affect the results. Secondl, this study did not analyze the number and volume of thyroid nodules, so its observation content was relatively single. At last, the representativeness of this study is limited due to the insufficient sample size. Notwithstanding its limitation, the present study could clearly indicate that there is a close association between microalbuminuria and TNs in T2DM. Next, we will conduct a multidimensional study of thyroid nodules to evaluate the predictive value of urinary microalbumin and further explore the internal mechanism between thyroid nodules and MAU through experiments.

## Figures and Tables

**Table 1:** Clinical and laboratory characteristics of study subjects.

Variables	Thyroid nodule group (*n* = 172)	Control group (*n* = 98)	*z* or *χ*^2^ values	*P* values
Sex				
Male, *n* (%)	90 (52.3)	69 (70.4)	8.432	0.004
Female, *n* (%)	82 (47.7)	29 (29.6)		

Age (years)	57 (50,65)	50 (44,60)	−3.763	<0.001

MAU				
Normal, *n* (%)	95 (55.2)	76 (77.6)	13.391	<0.001
Abnormal, *n* (%)	77 (44.8)	22 (22.4)		

RASi				
Yes, *n* (%)	73 (42.4)	25 (25.5)	7.740	0.005
No, *n* (%)	99 (57.6)	73 (74.5)		

u-WBC				
Normal, *n* (%)	169 (98.3)	98 (100)	0.506	0.477
Abnormal, *n* (%)	3 (1.7)	0 (0)		

u-RBC				
Normal, *n* (%)	164 (95.3)	98 (100)	3.219	0.073
Abnormal, *n* (%)	8 (4.7)	0 (0)		
TT3 (ng/mL)	0.85 (0.77, 0.96)	0.82 (0.73, 0.95)	−1.325	0.185
FT3 (pg/mL)	3.09 (2.81, 3.36)	3.19 (2.92, 3.44)	−1.529	0.126
TSH (uIU/mL)	1.67 (1.18, 2.57)	1.70 (1.25, 2.47)	−0.409	0.682
TT4 (ug/dL)	8.34 (7.45, 9.31)	7.78 (7.03, 8.81)	−2.497	0.013
FT4 (ng/dL)	0.89 (0.82, 0.98)	0.86 (0.77, 0.95)	−2.077	0.038
TPOAB (IU/mL)	0.50 (0.20, 1.45)	0.55 (0.20, 2.10)	−1.090	0.276
TGAB (IU/mL)	0.00 (0.00, 0.10)	0.00 (0.00, 0.20)	−1.814	0.070
SCR (umol/L)	56.0 (45.50, 69.50)	58.0 (45.0, 69.0)	−0.259	0.795
BUN (mmol/L)	4.70 (3.90, 5.50)	4.60 (3.80, 5.80)	−0.043	0.966
UA (umol/L)	285.0 (237.0, 358.0)	287.0 (230.0, 366.0)	−0.107	0.915
FPG (mmol/L)	7.30 (5.90, 9.90)	7.30 (6.00, 9.80)	−0.193	0.847
HDL (mmol/L)	0.95 (0.77, 1.13)	0.94 (0.82, 1.06)	−0.493	0.622
TC (mmol/L)	4.44 (3.71, 5.09)	4.23 (3.70, 5.12)	−0.764	0.445
LDL (mmol/L)	2.52 (2.01, 3.28)	2.42 (1.98, 3.34)	−0.081	0.935
TG (mmol/L)	1.58 (1.09, 2.50)	1.50 (1.05, 2.50)	−0.501	0.616
HBA1C (%)	9.21 (8.11, 10.48)	9.16 (7.99, 11.04)	−0.547	0.584
UALB (mg/L)	22.00 (10.0, 67.55)	11.15 (10.0, 25.8)	−3.491	<0.001

**Table 2:** Binary logistic regression analysis for predictors of thyroid nodule.

Items	*β* values	Wald values	*P* values	OR	95% CI
Sex	−0.862	8.651	0.003	0.422	0.238–0.750
Age	0.033	7.750	0.005	1.034	1.010–1.058
MAU	1.128	13.776	<0.001	3.090	1.703–5.605
FT4	2.462	6.089	0.014	11.732	1.660–82.930

**Table 3:** Correlation of thyroid nodules and predictors.

Variable	rs values	*P* values
Gender	0.177	0.004
Age (years)	0.229	<0.001
MAU	0.223	<0.001
FT4 (ng/dL)	0.127	0.038
TT4 (ug/dL)	0.152	0.012
UALB (ug/mL)	0.213	<0.001

**Table 4:** Ultrasound features of thyroid nodules.

Variables	MAU group (*n* = 77)	Non-MAU group (*n* = 95)	*χ* ^2^ values	*P* values
Echogenicity				
Hypoechoic, *n* (%)	27 (35.1)	43 (45.3)	1.833	0.176
Nonhypoechoic, *n* (%)	50 (64.9)	52 (54.7)		

Shape				
Taller-than-wide, *n* (%)	0 (0)	4 (4.2)	1.724	0.189
Wider-than-tall, *n* (%)	77 (100)	91 (95.8)		

Margins				
Irregular, *n* (%)	8 (10.4)	10 (10.5)	0.001	0.977
Regular, *n* (%)	69 (89.6)	85 (89.5)		

Echogenic foci				
Microcalcification, *n* (%)	10 (13.0)	16 (16.8)	0.493	0.483
Nonmicrocalcification, *n* (%)	67 (87.0)	79 (83.2)		

Scores of ultrasound				
High, *n* (%)	6 (7.8)	14 (14.7)	1.996	0.158
Low, *n* (%)	71 (92.2)	81 (85.3)		

## Data Availability

The data used to support the findings of this study are available from the corresponding author upon request.
